# State Medical Association of Illinois

**Published:** 1849-08

**Authors:** 


*State Medical Association, of Illinois.*

We have received a circular issued by our friend Dr. E. A. Guilbert, Secretary of the Ottawa Medico Chirurgical Association, inviting a co-operation of the several district societies throughout the state, in the formation of a State Association. The advantage of state organizations are so obvious, and so fully exemplified by experience, that we are sure the requisite efforts only are necessary to effect the object in the already great, and rapidly growing state of Illinois. The first of January, 1850, is designated as the time at which the convention is proposed to be held at the Capitol of the State. The Ottawa Medico-Chirurgical Society, we are pleased to learn, is in successful operation, and is effecting much good. We shall be glad to receive communications made to it, for publication in our columns, as suggested in the letter of the Secretary of the society.

---

JACKSON, MIGHIGAN, July 6, 1849.

Dr. FLINT—*Dear Sir:*—Having found the following mixture of great utility in the treatment of cholera morbus, and the various forms of irritation of the stomach and bowels, which is so prevalent in our State at this time, I am induced to send the formula to you, believing that it will be a matter of great convenience, if not an important article in the treatment of epidemic *cholera*, in its incipient stage.

| | |
|---|---|
| ℞.—Aq. Camphoræ, | 4℥ |
| Nitro muriatic acid, | 1ʒ |
| Tinc. Opi., | 2ʒ |
| Tannin. | 1℈ |
| Sal Alba, | 2℥ |

Mix—Dose.—1ʒ once in ten or fifteen minutes.

